# The effect of working memory training on test anxiety symptoms and attentional control in adolescents

**DOI:** 10.1186/s40359-024-01597-w

**Published:** 2024-02-27

**Authors:** Chunling Xu, Hua Wei

**Affiliations:** Department of Psychology, Suzhou University of Science and Technology, 99 Xuefu Road, Huqiu District, 215009 Suzhou, Jiangsu China

**Keywords:** Adolescent, Test anxiety, Attentional control, Working memory training, Intervention

## Abstract

**Objective:**

The percentage of adolescents with test anxiety is increasing rapidly. Working memory (WM) training has been demonstrated to reduce anxiety levels and enhance attentional control in individuals. Therefore, we investigated whether adaptive dual n-back WM training could lower test anxiety level and improve attentional control in adolescents.

**Methods:**

Forty adolescents were allocated to either adaptive dual n-back WM training (*n* = 21) or non-adaptive dual 1-back WM training (*n* = 19) for 10 days. The Test Anxiety Scale was applied to measure individuals’ test anxiety symptoms. The Attentional Control Scale (ACS), the flanker task, and the Go/Nogo task were used to measure attentional control.

**Results:**

Compared with the control group, the training group reported significantly relief of test anxiety symptoms; however, there were no significant differences between the two groups in pre-to-post changes in ACS scores or performance on the flanker task and Go/Nogo task.

**Conclusion:**

In sum, adaptive dual n-back WM training effectively reduced adolescents’ level of test anxiety but did not improve their attentional control.

## Introduction

Test anxiety refers to the symptoms produced in specific test-related situations, which can cause cognitive, physiological, emotional, and behavioral responses. Excessive worry about tests may lead to poor educational performance, low self-efficacy, and academic failure [1–3]. According to Putwain and Daly [4] and Thomas, Cassady [5], 15–20% of students reported experiencing a high level of test anxiety. China is renowned worldwide for its test-driven educational system, and test pressure is particularly high for adolescents in this education system; up to 30.8% of adolescents report suffering from high test anxiety [6]. With the advent of educational involution, test anxiety among adolescents has become more severe [7]. In addition, the effects of high levels of test anxiety are most evident in the adolescent stage and can lead to depression-related symptoms and other mental illnesses. Thus, it is imperative to develop effective interventions to reduce the level of test anxiety among Chinese adolescents.

Previous studies have demonstrated a significant association between test anxiety and working memory (WM). WM is not only an important system for temporary storage of information [8], but also a mechanism intimately tied to individuals’ attentional control (AC) [9, 10]. In the process of WM, constant AC resources are needed to capture task-related stimuli and suppress the interference of task-unrelated stimuli [9, 11]. However, anxious individuals have AC deficits [12–15], which makes it easier for them to be biased toward task-unrelated stimuli for which they cannot easily eliminate interference. Thus, attentional resources that should otherwise be put toward task-related stimuli are reduced [16–19]. Accordingly, individuals with test anxiety are more likely to be disturbed by task-unrelated stimuli [20, 21], which can lead to a decline in WM [22, 23]. According to processing efficacy theory [24], individuals who worry excessively will take up major attentional resources and experience a decline in their WM [25]; this will have a significant negative impact on individuals’ anxiety levels [9, 26, 27].

Therefore, enhancing individuals’ WM may improve their AC and reduce anxiety-related symptoms [27, 28]. WM can be increased with adaptive dual n-back WM training, which is one of the most popular interventions for improving individuals’ WM [29–31]. In adaptive dual n-back WM training, participants need to remember two stimuli simultaneously (e.g., sound, color, shape, location, etc.), which are shown randomly, and compare the current stimulus with the previous n-number stimuli. As the level of n-back increases, participants have to memorize more stimuli, which could increase their WM [29, 32], further improve their AC [16, 17, 28, 33], and reduce anxiety-related symptoms [32, 34, 35], including test anxiety [36].

In recent years, many researchers have explored this field, but their conclusions have been inconsistent. Some recent studies have shown the benefits of adaptive dual n-back WM training in improvements in AC and the reduction of anxiety-related symptoms for individuals [29, 37]. For example, Sari, Koster [37] used an adaptive dual n-back WM training task, which lasted for 15 days, to investigate the effects of training on AC in high trait anxiety undergraduates. The results showed that, after training, the AC of undergraduates with high trait anxiety improved and their trait anxiety level also reduced [37]. However, some studies have indicated that adaptive dual n-back WM training can enhance individuals’ AC but cannot reduce anxiety or depression symptoms [30, 38, 39]. For example, in a study by Ducrocq, Wilson [38], participants underwent either adaptive dual n-back WM training or a non-adaptive version for 10 days; adaptive WM training enhanced training groups’ WM capacity, which was more beneficial to improving their AC, but did not change their anxiety level. In addition, some studies have shown that adaptive dual n-back WM training significantly reduced individuals’ anxiety and worries level, but did not produce any differential effects on AC [40, 41]. Hotton, Derakshan [41] conducted adaptive dual n-back WM training over 15 days and performed pre-test, post-test, and follow-up tests. The results displayed improvements in anxiety and worries symptoms, but individuals’ AC remained the same.

Based on the above studies, we could argue that WM training can effectively lower the level of test anxiety and improve AC in adolescents. Although this is theoretically feasible, outcomes have been inconsistent. In particular, there have been few studies on interventions for individuals with test anxiety, especially WM training for adolescents with test anxiety. In a study by Minihan, Samimi [36], college students with test anxiety symptoms received 20 days of WM training, after which their test anxiety symptoms were lower and their emotional regulation was greater. Wei, De Beuckelaer [39] employed WM training task to conduct 20 days of training for college students with high test anxiety; the effects of the training only led to a significant improvement in the inhibition ability of individual dominant responses at the neurophysiological level but could not change the level of test anxiety. However, as the participants in these two studies were adults, we do not know whether WM training can reduce test anxiety levels or improve AC in adolescents.

Given the importance of relieving test anxiety symptoms in adolescents, we aimed to confirm the impact of adaptive dual n-back WM training on enhancing AC and reducing the level of test anxiety in adolescents. Adolescents were selected to participate in either adaptive dual n-back WM training or non-adaptive dual 1-back WM training for 10 days. By comparing changes in the level of test anxiety (measured by the Test Anxiety Scale [TAS]) and AC (measured by the Attentional Control Scale [ACS], the flanker task, and the Go/Nogo task) between the training and control groups from pre- to post-tests, it was possible to investigate the effects of WM training on each adolescent’s test anxiety level and AC.

Based on previous studies [34, 37], we hypothesized that the adaptive dual n-back WM training would significantly improve AC and lower the level of test anxiety in adolescents. Specifically, we expected that, in comparison with the control group, the adaptive dual n-back WM training would improve behavioral performance on flanker and Go/Nogo tasks, as well as enhance the scores on the ACS, and led to a reduction in TAS scores.

## Method

### Participants

**Fig. 1:**
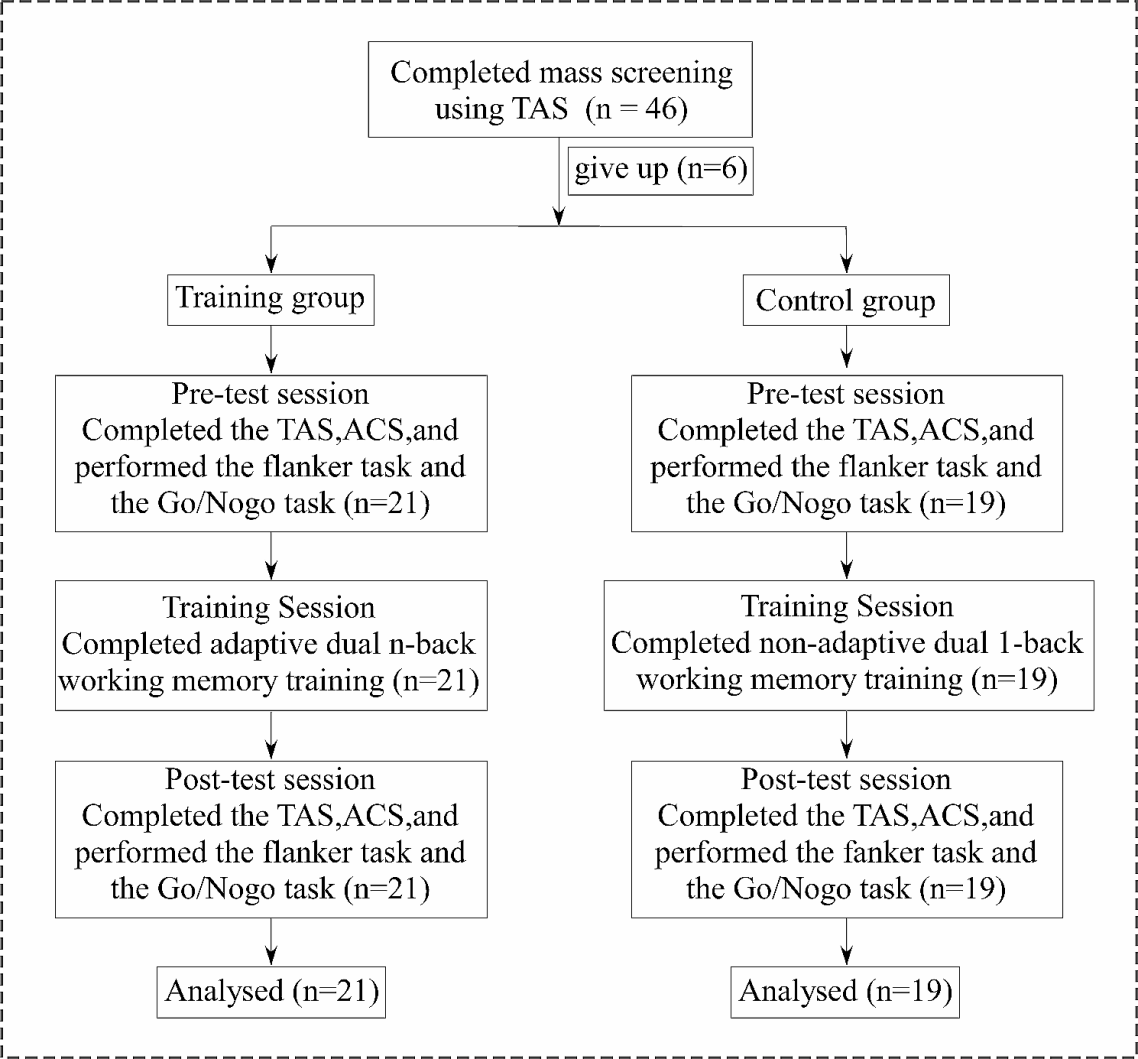
Study flowchart Note: TAS, Test Anxiety Scale; ACS, Attentional Control Scale

The sample size used in our study was determined by G*Power software [42]. According to prior studies [43], we determined a moderate expected effect size of f = 0.25, corresponding statistical power level of 1 − β = 0.80 (α = 0.05), and a minimal sample size of 17 in each group, so we needed 34 participants. In the present study, initially, 46 participants were selected using simple random sampling. They ranged in age from 11 to 17 years old and were from a middle school in China. Participants were randomly allocated to the training and control group, and using a minimization principle to balance the two groups in terms of age, and scores of test anxiety. As shown in Fig. 1, six participants completed the screening questionnaire but dropped out before the pre-test task. In total, 40 participants persisted to the final post-test task, with 21 participants (10 women, M = 14.05 years, SD = 1.77) completing adaptive dual n-back WM training and 19 participants (9 women, M = 13.95 years, SD = 2.12) completing non-adaptive 1-back WM training. There were no significant differences between those groups in terms of age *t*(38) = 0.68, *p* =.51, Cohen’s d = 0.16, and scores of test anxiety, *t*(38) = 0.09, *p* =.93, Cohen’s d = 0.02.

All participants voluntarily took part in the experiment, as evidenced by their written informed consent, and they completed the entire experiment during the winter vacation. After the experiment, each participant was given RMB 100 (about USD 14.5) as a reward. This study was approved by the Ethics Committee of the Department of Psychology at Suzhou University of Science and Technology. Experiments were carried out according to the corresponding requirements.

### Self-report scales

#### TAS

The TAS is a 37-item, unidimensional questionnaire that measures symptoms of test anxiety. Participants answered with either *agree* (score 1 point) or *disagree* (score 0 point) responses, with scores ranging from 0 to 37, higher scores indicated higher levels of test anxiety [44]. The Chinese version of the TAS was refined by Caikang Wang from the original version constructed by Irwin G. Sarason [44, 45], which was applicable to China and demonstrated high internal consistency (Cronbach’s alpha = 0.87, *n* = 1966) [46].

#### ACS

The ACS is a two-dimensional questionnaire with 20-item that combined the attentional focusing and shifting scales to assess AC, with a Likert scale ranging from 1 (*not consistent with me at all*) to 4 (*consistent with me very much*) [47]. The scores range from 20 to 80, higher scores indicate greater AC of the individuals. The Chinese version of the ACS was refined by Huizi Zhang, which was applicable to China and demonstrated high internal consistency (Cronbach’s alpha = 0.83, *n* = 420) [48].

### Behavioral tasks

#### The flanker task

**Fig. 2:**
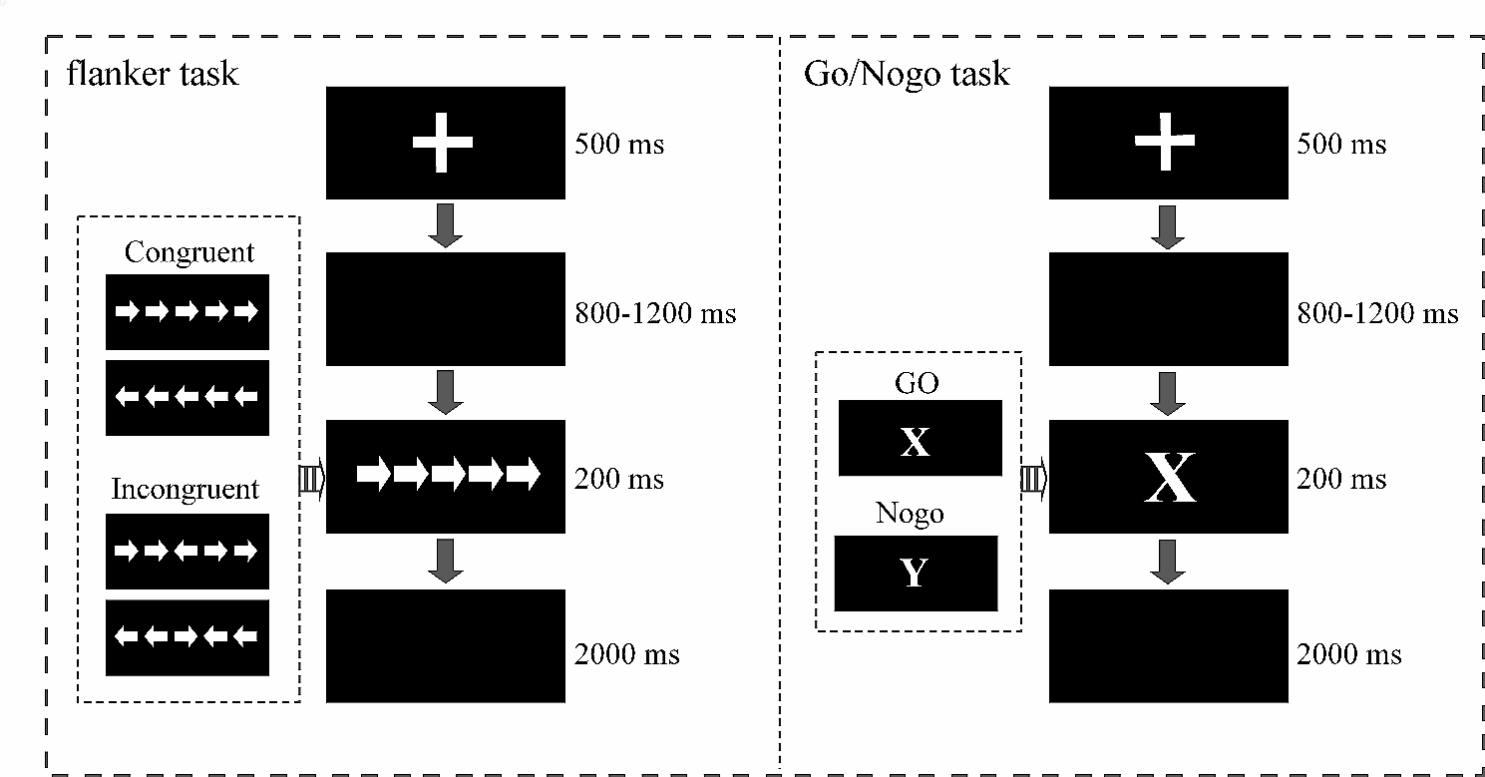
Schematic representation of the flanker task and the Go/Nogo task

We used Wei, De Beuckelaer [39] modified version of the flanker task originally conceived by Eriksen and Eriksen [49]. As shown in Fig. 2, the task began with a white cross appearing in the center of a black screen for 500 ms. Then, a blank screen was shown for a random period between 800 and 1200 ms. Next, a set of five parallel arrows appeared in the middle for 200 ms. Participants were instructed to look at the direction of the most central of the five arrows and press the *F* key if it pointed left or the *J* key if it pointed right. Finally, a blank screen was shown for 2000 ms. Then the next trial began.

This task contained one practice block and two experimental blocks. The practice block was presented first and included 20 trials. After the practice period, participants could choose whether to continue practicing or to start the experimental task. The experimental blocks included 100 trials in each block. In half of the trials in each block, both the target stimulus (the most central arrow) and the distractor stimuli (the two side arrows) were shown in the same direction, which was in the congruent condition (→→→→→; ←←←←←). In the other half of the trials, the target stimulus and the distractor stimuli pointed in opposite directions, which was in the incongruent condition (←←→←←; →→←→→). Both the congruent and incongruent conditions appeared randomly.

#### The Go/Nogo task

We used Wei, De Beuckelaer [39] modified version of the Go/Nogo task originally conceived by Simson, Vaughan Jr [50]. As displayed in Fig. 2, the task began with a white cross appearing in the center of a black screen for 500 ms. Then, a blank screen was shown randomly for 800–1200 ms. Afterward, a letter stimulus (*X* or *Y*) appeared in the middle of the screen for 200 ms. Participants pressed the space bar when the letter *X* (target stimulus) appeared but made no response if the letter *Y* (distractor stimulus) appeared. Participants needed to look at the letter and respond quickly. Finally, a blank screen was shown for 2000 ms. The next trial began.

This task contained one practice block and two experimental blocks, practice block presented first and included 20 trials, after which participants could choose whether to continue practicing or to start the experimental task; the experimental blocks included 120 trials in each block. Ninety stimuli that required a response and 30 stimuli that did not were included in each experimental block, and both stimulus conditions appeared randomly.

### The WM training task

#### The adaptive dual n-back WM training task

**Fig. 3:**
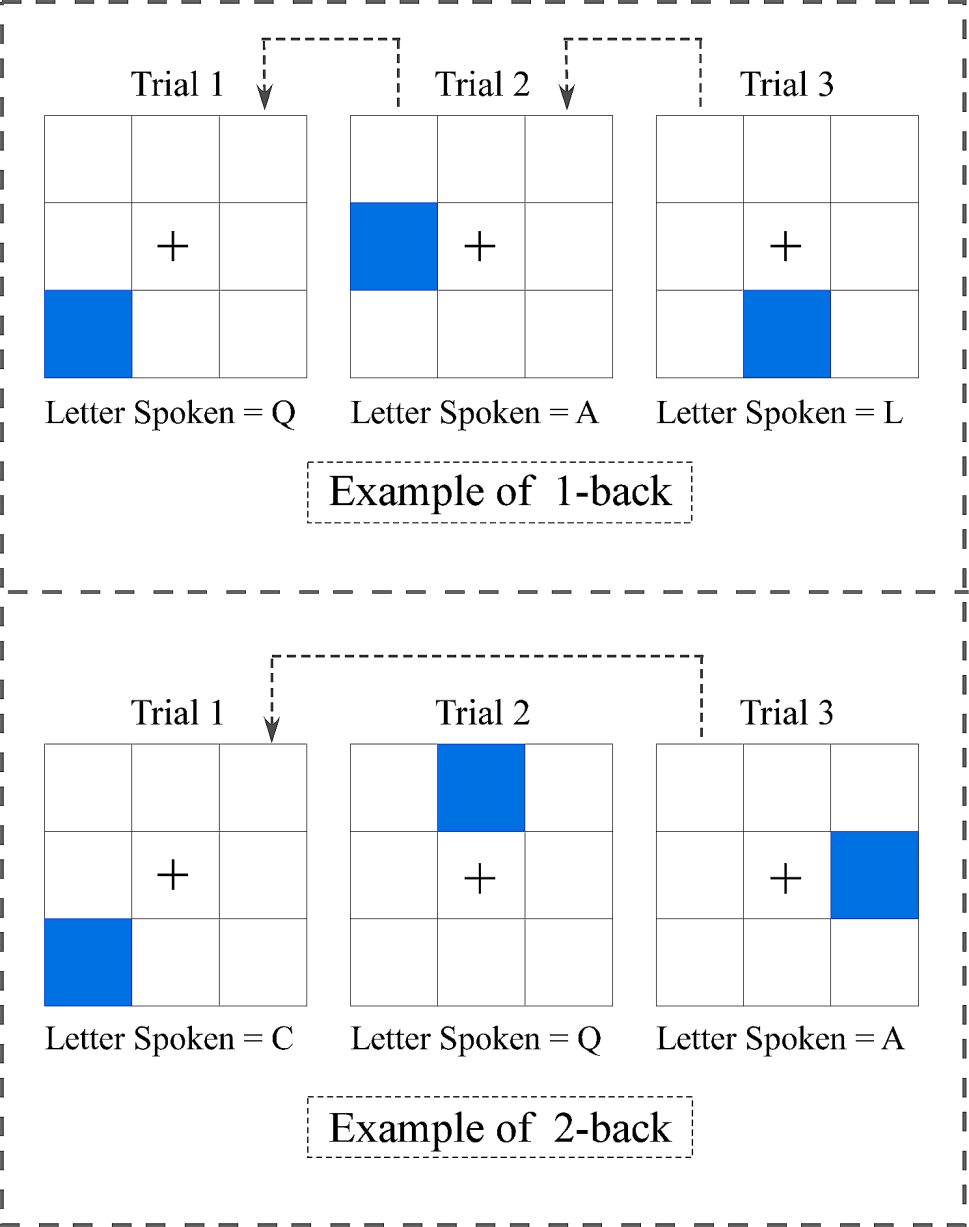
The dual n-back WM training task. An example of the 1/2-back trial

In this study, we adopted an adaptive dual n-back WM training task to train participants’ WM, similar to that of Sari, Koster [37]. As displayed in Fig. 3, the task consisted of a 3 × 3 grid, blue squares, and spoken letters. Each trial began with a cross-fixation point appearing in the center of the nine squares, immediately after the cross-fixation point disappeared, a blue square appearing randomly in one of the remaining eight squares, and a letter being simultaneously announced. Participants needed to memorize the location of the blue square and what letter was spoken (500 ms). After 2500 ms, another trial stimuli pair was presented. As soon as the blue square appeared and the letter was spoken, participants must immediately recall the trial stimuli pair returned n times from the current trial, and judge whether the stimuli pair of the two trials match. If the blue square location matched, participants pressed the *A* key; if the spoken letters matched, they pressed the *L* key; if both matched, they pressed both keys; and if neither matched, they made no response. The numbers of the position and letter matches were randomized for each block. Participants began at the dual 2-back task. If accuracy on positions and letters was 80% or greater, the level of the task would increase to 3-back. If accuracy fluctuated between 50% and 79%, the task would stay at the same level. If accuracy was below 50% three times in a row, the task would be made easier. As their accuracy rate increased, participants could reach continuously higher levels. Each training session lasted approximately 40 min and consisted of 20 blocks, each containing 20 + n trials.

#### The non-adaptive dual 1-back WM training task

Consistent with the adaptive dual n-back WM training task, participants had to remember the random location of a blue square and an arbitrary letter simultaneously announced. Unlike in the adaptive version, the task remained at the 1-back level, i.e., participants only practiced the 1-back task. Specifically, participants needed to compare the current two stimuli with the trial stimuli pair returned once from the current trial. They used the same keys to record matches as in the adaptive version. There were 20 blocks in each session, and 21 trials in each block. The whole session lasted 25 min.

### Procedure

With the permission of their parents and teachers, participants completed the TAS screening questionnaire. Based on their TAS scores and age, participants were allocated to the adaptive dual n-back WM training or non-adaptive dual 1-back WM training. As shown in Fig. 1, in the formal experiment we told participants that this was a valid memory test and that they needed to be careful. First, the participants completed the TAS and ACS on a mobile device. They then completed the flanker task and the Go/Nogo task on a specific computer. The entire process lasted 35–45 min. During the training stage, the Brain Workshop was installed on all participants’ computers to inform them of the corresponding training requirements. The training group completed adaptive dual n-back WM training in which the level changed according to their performance. By contrast, the control group completed non-adaptive dual 1-back WM training in which the level did not change. Both groups were asked to complete the task for five days each week, and the whole training lasted two weeks. Participants sent screenshots to the researcher at the end of each training session. To ensure that the participants would not drop out, we met with them regularly to offer encouragement and gifts and we sought the help of their parents. After two weeks, we invited the participants back to the lab for the post-test task, which was the same as the pre-test task. The participants received a predetermined financial reward.

### Statistical analysis

All statistical analyses involved in this study were conducted in SPSS (version 25.0). In the WM task, the mean n-back level on the first and last day of the training group was analyzed by the paired sample t-test (two-tailed), and the same method was applied to analyze the accuracy on the first and last day of the control group, and we computed the Cohen’s d to estimate the effect size of the paired sample t-test (two-tailed). Referring to Sari, Koster [37], we used interference scores (subtracting the accuracy and response times [RTs] in congruent trials from the accuracy and RTs in incongruent trials) to measure outcomes of the flanker task. Based on previous studies [51], participant’s scores on the TAS, ACS, interference scores of the RTs and accuracy in the flanker task, scores of the Go RTs, Go accuracy and Nogo accuracy in the Go/Nogo task were analyzed separately using a two-way mixed repeated-measures analysis of variance (ANOVA) with time (pre- and post-) as within-subject factor, and with group (adaptive WM training group and control group) as between-subject factor. All of the data were consistent with the sphericity assumption. Finally, we used Spearman’s correlation analyses (two-tailed) to analyzed the correlations of WM training gains (subtracting the mean n-back level on the first day from the mean n-back level on the last day) with reductions in test anxiety levels and improvements in AC. We computed the partial eta-squared (η_p_^2^) for ANOVAs to estimate the effect size of the F tests. An alpha level of 0.05 was adopted as criterion for statistical significance in this study.

## Results

### Performance on the training and control dual n-back WM tasks

**Fig. 4:**
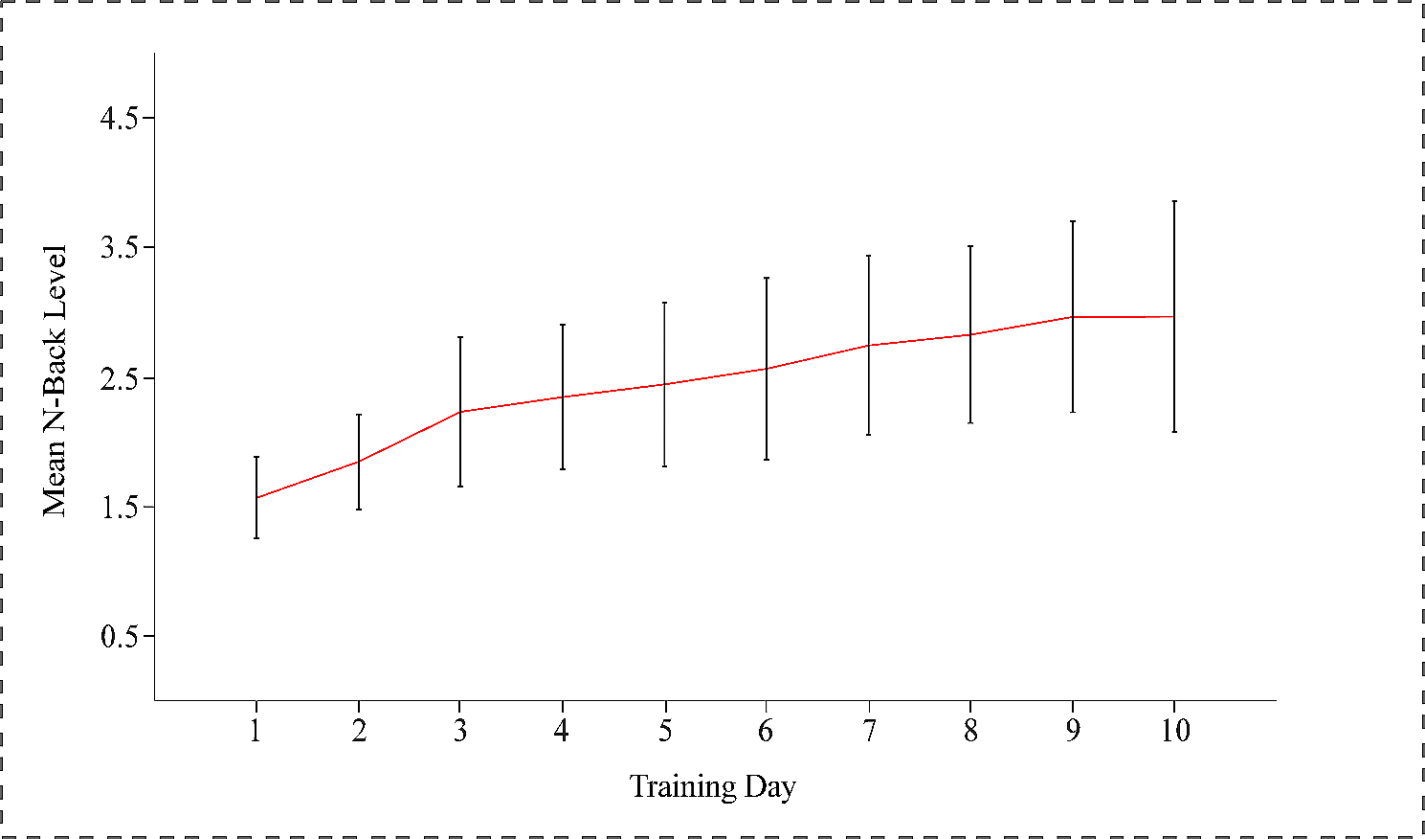
Performance of the training group over time on the dual n-back WM training task. Error bars show the standard deviation

Fig. 4 shows the mean n-back level achieved by the training group during the two weeks. The WM of the training group improved due to adaptive dual n-back WM training, as evidenced by a higher mean n-back level on the last day of training (M = 2.96, SD = 0.89) relative to the first day (M = 1.56, SD = 0.31), *t*(20) = 7.55, *p* <.01, Cohen’s d = 1.65. In the control group, the scores improved from the first day of training (M = 75.68%, SD = 11.73%) to the last day (M = 89.00%, SD = 10.03%), *t*(18) = 4.65, *p* <.01, Cohen’s d = 1.07.

### Self-reported symptomatology

Table 1 shows the descriptive statistics for the pre- and post-test scores of each group on the TAS and ACS.

**Table 1:** Pre- and post-test self-reported scores and behavioral results (M ± SD) for all conditions

		Training group		Control group		
		Pre-	Post-	Pre-		Post-
TAS	Sum score	19.95 ± 7.07	17.10 ± 7.66	19.63 ± 6.86	20.05 ± 7.38	
ACS	Sum score	47.52 ± 7.22	48.57 ± 7.36	49.32 ± 5.20	48.37 ± 4.95	
Go/Nogo	GO RTs	486.01 ± 100.65	484.79 ± 111.13	544.94 ± 86.69	590.63 ± 91.15	
	GO accuracy	95.42% ± 7.21%	94.81% ± 6.48%	92.98% ± 6.67%	88.65% ± 12.69%	
	Nogo accuracy	10.79% ± 9.20%	8.57% ± 12.78%	10.09% ± 10.52%	6.84% ± 8.68%	
flanker	RTs interference scores	82.72 ± 34.91	56.87 ± 21.67	94.81 ± 41.88	80.93 ± 38.58	
	accuracy interference scores	-4.19% ± 5.33%	-2.62% ± 3.54	-4.21% ± 4.37%	-1.68% ± 2.11%	

Note: TAS, Test Anxiety Scale; ACS, Attentional Control Scale; interference scores, subtracting the accuracy and response times (RTs) in congruent trials from the accuracy and response times (RTs) in incongruent trials

#### TAS

A two-way mixed ANOVA showed no significant main effect of time, *F*(1,38) = 2.84, *p* =.10, η_p_^2^ = 0.07, or group, *F*(1,38) = 0.37, *p* =.55, η_p_^2^ = 0.01; the interaction effect between time and group was significant, *F*(1,38) = 5.15, *p* =.03, η_p_^2^ = 0.12. Further analysis revealed that, (1) for the training group, the TAS scores were significantly lower in the post-test (M = 17.10, SD = 7.66) than in the pre-test (M = 19.95, SD = 7.07, *p* <.01), whereas the control group did not differ from the post-test period (M = 20.05, SD = 7.38) to the pre-test (M = 19.63, SD = 6.86, *p* =.69); (2) In the pre-test, there were no significant differences between the training group (M = 19.95, SD = 7.07) and the control group (M = 19.63, SD = 6.86, *p* =.89); in the post-test, there were no significant differences between the training group (M = 17.10, SD = 7.66) and the control group (M = 20.05, SD = 7.38, *p* =.22).

#### ACS

A two-way mixed ANOVA indicated no significant main effect of time, *F*(1,38) = 0.003, *p* =.96, η_p_^2^ < 0.01, or group, *F*(1,38) = 0.19, *p* =.66, η_p_^2^ = 0.01. There was no significant interaction effect between time and group, *F*(1,38) = 1.30, *p* =.26, η_p_^2^ = 0.03.

### The flanker task

Table 1 lists the interference scores of the RTs and accuracy for the flanker task.

#### RTs

A two-way mixed ANOVA revealed a main effect of time, *F*(1,38) = 7.69, *p* =.01, η_p_^2^ = 0.17, indicating that the RTs’ interference scores were significantly smaller in the post-test (M = 68.30, SD = 32.80) than in the pre-test (M = 88.46, SD = 38.36) and for group as well, *F*(1,38) = 4.65, *p* =.04, η_p_^2^ = 0.11, implying that the RTs’ interference scores were significantly smaller in the training group (M = 69.79, SD = 21.97) than in the control group (M = 87.87, SD = 30.70); there were no significant interaction effect between time and group, *F*(1,38) = 0.70, *p* =.41, η_p_^2^ = 0.02.

#### Accuracy

A two-way mixed ANOVA indicated a main effect of time, *F*(1,38) = 6.63, *p* =.01, η_p_^2^ *=* 0.15, suggesting that the accuracy interference scores were significantly improved in the post-test (M = 2.18%, SD = 2.95%) than in the pre-test (M = 4.20%, SD = 4.84%). There was no significant main effect of group, *F*(1,38) = 0.21, *p* =.65, η_p_^2^ *=* 0.01. The interaction effect between time and group were also not significant, *F*(1,38) = 0.36, *p* =.55, η_p_^2^ = 0.01.

### The Go/Nogo task

Table 1 displays the results of the RTs and accuracy for the Go/Nogo task.

#### Go RTs

A two-way mixed ANOVA showed a main effect of group, *F*(1,38) = 9.82, *p* <.05, η_p_^2^ = 0.21, implying that the RTs’ scores were significantly smaller in the training group (M = 485.40, SD = 86.57) than in the control group (M = 567.79, SD = 78.96). There was no significant main effect of time, *F*(1,38) = 1.78, *p* =.19, η_p_^2^ = 0.05, and the interaction effect between time and group were not significant, *F*(1,38) = 1.99, *p* =.17, η_p_^2^ = 0.05.

#### Go accuracy

A two-way mixed ANOVA revealed no significant main effect of time, *F*(1,38) = 2.19, *p* =.15, η_p_^2^ = 0.05, or of group, *F*(1,38) = 4.04, *p* >.05, η_p_^2^ = 0.10. The interaction effect between time and group was also not significant, *F*(1,38) = 1.24, *p* =.27, η_p_^2^ = 0.03.

#### Nogo accuracy

Two-way mixed ANOVA demonstrated no significant main effect of time, *F*(1,38) = 1.77, *p* =.19, η_p_^2^ = 0.04 or of group, *F*(1,38) = 0.22, *p* =.64, η_p_^2^ = 0.01. The interaction effect between time and group was not significant, *F*(1,38) = 0.06, *p* =.81, η_p_^2^ < 0.01.

### Correlations between WM training gains and the scores of self-reported scales and outcomes of behavioral tasks

#### Correlations between WM training gains and changes in self-reported scales (the scale scores from the post-test — the scale scores from the pre-test) in the training group

The results of the correlation analysis showed no significant correlations between the WM training gains and changes in TAS scores (*r*(19) = 0.09, *p* =.70) or changes in ACS scores (*r*(19) *=* 0.23, *p* =.31).

#### Correlations between WM training gains and outcome changes of the flanker task (interference scores from the post-test–— interference scores from the pre-test) in the training group

The results of the correlation analysis indicated no significant correlations between WM training gains and the changes in interference scores of the RTs (*r*(19) *=* 0.10, *p* =.68). There were significant correlations between the WM training gains and changes in the accuracy interference scores (*r*(19) = 0.51, *p* =.02).

#### Correlations between WM training gains and outcome changes of the Go/Nogo task (scores from the post-test — scores from the pre-test) in the training group

The results of correlation analysis revealed no significant correlations between WM training gains and the score changes of Go RTs (*r*(19) *=* 0.14, *p* =.53), the score changes of Go accuracy(*r*(19) *=* 0.07, *p* =.76), or the score changes of Nogo accuracy (*r*(19) = 0.18, *p* =.43).

## Discussion

We explored whether adaptive dual n-back WM training can greatly enhance AC and lower test anxiety symptoms in adolescents. An important finding of this study is that adaptive dual n-back WM training led to a significant reduction in the level of test anxiety in adolescents, which manifested as a significant decline in TAS scores for the training group in comparison with the control group. Inconsistent with our hypothesis, the results failed to demonstrate that adaptive dual n-back WM training enhanced flanker task outcomes, Go/Nogo task outcomes, and ACS scores in the training group in comparison with the control group.

Little research has been conducted on whether WM training can reduce an individual’s test anxiety symptoms, and results have been inconsistent. In a study by Minihan, Samimi [36], participants completed 20 days of WM training; results showed that the intervention reduced individuals’ test anxiety levels. However, in a study by Wei, De Beuckelaer [39], WM training did not significantly reduce the level of test anxiety in high-test-anxiety individuals. Furthermore, we found inconsistent outcomes regarding whether the WM training can reduce social, trait, and other anxiety symptoms. For example, Zhao, Dang [43] found that WM training could effectively lower college students’ social anxiety levels over 20 days, in comparison with the control group. Sari, Koster [37] and Nazari, Fazilat-Pour [52] conducted studies on college students and adolescents, respectively, and found that WM training can significantly reduce trait anxiety levels. In a study by de Voogd, Wiers [30] of an eight-day intervention, anxiety and depression levels did not decline significantly in the training group in comparison with the control group. In the current study, adaptive dual n-back WM training significantly lowered the level of test anxiety in adolescents, which may be due to their high plasticity as they can enhance their emotional regulation ability after short-term intervention [34]. As in the study of Beloe and Derakshan [34], adaptive dual n-back WM training was found to relieve adolescents’ anxiety and depression-related symptoms and the effects lasted one month. And, we selected adolescents with test anxiety in the current study at random, rather than adolescents with high test anxiety, which further supports the feasibility of WM training as an intervention for adolescents with test anxiety. Given that our study covered all adolescents, future research should further investigate the results of WM training in adolescents at various phases.

We found, however, that adaptive dual n-back WM training failed to enhance the task performance of adolescents on the flanker and Go/Nogo tasks and their scores on the ACS when compared to the 1-back control group. In terms of the flanker task, there was no significant difference between the groups after training, and both groups experienced improvements in the accuracy interference scores and reductions in the RT interference scores. However, the interaction between time and group was not significant. This outcome resembles the effects observed in a sample of individuals with high test anxiety [39] and worries [31, 41]. There are several explanations for this, one of which is that both groups exhibited an increase in WM performance after two weeks of training. This may have led to similar improvements in both groups on the flanker task. While the use of non-adaptive WM training as a control group in prior research was a common method of control group setting in previous studies, we assumed that this control group setup would have no training effects on individuals’ WM abilities. However, our study does not seem to support this conclusion. Thus, although most research designs tend to use a non-adaptive group as a control, whether such an experimental design is perfect needs to be further explored. Another likely reason is that WM training may impact on the AC of high-test-anxiety individuals only at the brain level, with no effect at the behavioral level. For example, in a study by Wei, De Beuckelaer [39] involving 20 days of WM training, college students’ performance in the flanker and Go/Nogo tasks did not significantly change in comparison with the control group, but the electroencephalography data of individuals while performing the Nogo task showed the effect of the training.

It is well known that test anxiety not only significantly affects each individual’s outcome on tests and academic performance [53], but also has the potential to lead to clinical anxiety [2] and depression-related symptoms [54], which have a negative impact on the health of individuals. However, according to research conducted abroad, 25% of students experience high levels of test anxiety [5] compared to more than one-third of middle school students in China, where the occurrence of test anxiety is rising annually [6]. According to Ciobotaru, Jefferies [31], adaptive dual n-back WM training reduced participants’ anxiety and depression-related symptoms. However, the impact of adaptive dual n-back WM training on test anxiety is inconsistent, and additional empirical research is required to prove this hypothesis. Notwithstanding, for adolescents, the training effects of adaptive WM training on test anxiety has yet to be proven in this age group. This study was the first to test anxiety symptoms in adolescents, and the results were impressive as we found reductions in the level of test anxiety in adolescents following adaptive dual n-back WM training. According to Beloe and Derakshan [34], adaptive WM training has the important potential to have a lasting effect on adolescents’ anxiety and depression-related symptoms. These results provide further evidence that adaptive dual n-back WM training is an effective way to reduce anxiety symptoms in adolescents. Thus, adaptive dual n-back WM training is a feasible long-term mechanism to reduce test anxiety levels in adolescents.

There were also some limitations in our study. First, although our sample size was calculated based on G*Power, it may be beneficial to have a larger sample size in future studies. Insufficient sample size may inhibit the identification of post-test differences between groups, especially for the outcomes of behavioral tasks [31, 37]. Second, we did not conduct a follow-up survey, and so cannot provide insights on whether the observed positive effects of adaptive dual n-back WM training were lasting. Hence, future research should employ a larger sample size and include follow-up tests to confirm the intervention effects of adaptive dual n-back WM training on adolescents’ test anxiety and AC.

In sum, we found significant reductions in the level of test anxiety in adolescents following 10 days of adaptive dual n-back WM training, but no improvements in AC in the training group in comparison with the control group. The present study offers preliminary evidence for the alleviating effect of adaptive dual n-back WM training on test anxiety symptoms in adolescents. Given the severity of test anxiety in adolescents [6], future research should explore more effective interventions for adolescents’ test anxiety symptoms.

## Data Availability

The dataset supporting the conclusions of this article is available in the *Psychological Science Data Bank* repository. 10.57760/sciencedb.psych.00151.

## Abbreviations

WM: Working memory
AC: Attentional control
ACS: The Attentional Control Scale
TAS: The Test Anxiety Scale
